# Cardiovascular Implications in Idiopathic and Syndromic Obesity in Childhood: An Update

**DOI:** 10.3389/fendo.2020.00330

**Published:** 2020-06-09

**Authors:** Maurizio Delvecchio, Carmela Pastore, Federica Valente, Paola Giordano

**Affiliations:** ^1^Metabolic Disorders and Diabetes Unit, “Giovanni XXIII” Children Hospital, AOU Policlinico di Bari, Bari, Italy; ^2^“B. Trambusti” Pediatric Unit, “Giovanni XXIII” Children Hospital, AOU Policlinico di Bari, Bari, Italy; ^3^Erasme Hospital, Université Libre de Bruxelles, Brussels, Belgium

**Keywords:** pediatric obesity, syndromic obesity, idiopathic obesity, cardiovascular disease, Prader–Willi syndrome, metabolic syndrome

## Abstract

Childhood obesity is a modern worldwide epidemic with significant burden for health. It is a chronic metabolic disorder associated with multiple cardiovascular risk factors such as dyslipidemia, hypertension, stroke, and insulin resistance. Many obese adolescents remain obese into adulthood, with increased morbidity and mortality. As childhood obesity is a risk factor for adult obesity, the childhood obesity-related disorders account for an increased risk of cardiovascular consequences in adults, in addition to the effects already exerted by the fat mass in adulthood. Several papers have already described the cardiovascular implications of idiopathic obesity, while few data are available about syndromic obesity, due to the small sample size, not homogeneous phenotypes, and younger age at death. The aim of this mini-review is to give a comprehensive overview on knowledge about cardiovascular implications of idiopathic and syndromic obesity to allow the reader a quick comparison between them. The similarities and differences will be highlighted.

## Background

Childhood obesity is a worldwide epidemic and a significant burden for health. In the WHO countries, one third of children is overweight or obese, and in the majority of European countries, above 30 and 10% of 5–19-year-old subjects are overweight or obese (1). Worryingly, about 60% of overweight prepubertal children become overweight in early adulthood, with the risk of developing noncommunicable diseases like hypertension, type 2 diabetes (T2D), metabolic syndrome (MetS), and cardiovascular diseases (CVDs) (2). Altogether, they account for ~77% of the burden of disease and almost 86% of premature mortality (1), with large economic and social impacts exacerbated by the early age at onset. Prevention strategies are needed.

The American Heart Association suggests that primary prevention should begin in childhood, when eating habits are acquired, achieving and maintaining a healthy diet (3). The role of nutritional education has been reevaluated, so that the WHO's Health Promoting Schools framework promotes interventions regarding lessons over preparing fruit/vegetable-based meals and snacks, lessons for teachers, promotion of physical activity, and reduction of sugary drinks. Many countries adopted strategies to fight obesity with short-term results, but long-term strategies are necessary because prevention is the only way to avoid its consequences (1, 4). In this view, prevention is important also for those workers who may receive less protection (5).

Childhood obesity can be classified into exogenous and endogenous: the former is caused by a chronic imbalance between energy intake and expenditure (idiopathic obesity) and the latter by endocrine, genetic, or syndromic disorders. In 97–98% of cases, obesity is idiopathic and related to lifestyle, eating habits, genetics, environment, and metabolism (Supplementary Table 1). The underlying mechanisms for cardiovascular implications appear different to some extent between idiopathic and syndromic obesity.

In this mini-review, we aimed to summarize the recent findings about the cardiovascular implications of childhood obesity to give a proper overview to the reader. We reported on idiopathic and syndromic obesity to give an easy and quick update of similarities and differences between them.

### Obesity Diagnosis in Childhood

The definition of pediatric obesity is heterogeneous, and there is still a need to standardize the measures and to establish comparable cutoff points between different populations.

Body fat mass can be estimated by several methods, such as dual-energy X-ray absorptiometry, bioelectrical impedance analysis, air displacement plethysmography, stable isotope dilution, magnetic resonance imaging, and skinfold thickness (6). However, the percentage of body fat is not routinely evaluated in clinical practice to diagnose obesity.

Body mass index (BMI) is the most frequently used parameter and tightly correlates with fat mass, despite some limitations (i.e., it does not distinguish between lean and fat mass nor consider the differences of body fat percentage across ethnicities) (7). International cutoffs for childhood obesity were established by the International Obesity Task Force by the LMS method.

Waist circumference (WC) may be used for the follow-up, but it lacks standard for children (7). A derivative of WC is the waist–hip ratio, an index of central adiposity well correlated with the CVD risk. Neck circumference is another index to evaluate obesity, which well correlates with BMI and WC (8). Wrist circumference correlates independently with MetS, left ventricular hypertrophy, and adipose tissue inflammation. Its role in predicting cardiometabolic risk (CMR) should be interpreted cautiously, and further cohort studies are needed (9, 10). Basically, indexes of visceral fat are predictive of cardiovascular and metabolic diseases.

## Methods

A systematic literature review was performed using the PRISMA guidelines. Two authors (CP and MD) independently performed a PubMed search to identify the pertinent papers updated to 15th February 2020 using the following MeSH terms: “pediatric obesity” AND “cardiovascular disease” OR “complications.” For syndromic obesity, “pediatric obesity” was replaced by the name of each syndrome. We searched for reviews, clinical trials, and observational studies about idiopathic obesity. Regarding syndromic obesity, we considered also case reports and case series. The only restriction criterion was full-text English manuscripts. Potentially relevant papers were evaluated by checking titles and abstracts, and all eligible studies were retrieved. Additional papers were identified by a manual search of the references from the retrieved articles.

## Idiopathic Obesity

Cardiovascular implications of childhood obesity are mediated by two main mechanisms: cardiovascular and metabolic. Their interplay creates a vicious circle (Supplementary Figure 1) that burdens the cardiovascular system leading to CVDs such as atherosclerosis, increased risk of coronary heart disease, and stroke with premature death (11).

### Cardiometabolic Risk

Cardiovascular and metabolic alterations may start as early as during adolescence. BMI is an independent predictor of coronary heart disease in adulthood, but a distinct pediatric threshold associated with increased CMR cannot be established. Increase in BMI before puberty, even within normal limits and persistent during puberty, results in increased prevalence of T2D, hypertension, coronary heart disease, and dyslipidemia as compared to a normal-weight age-matched population (12). Recently, Li et al. (13) explained the association between BMI trajectories and CMR in young children, showing that a rapid increment of BMI increases CMR, largely driven by WC.

We can classify the cardiovascular alterations in vascular damage and increase in preload while their effects in structural and functional ones on vessels and heart.

### Vessel Damage

Endothelial dysfunction and arterial stiffness occur with aging, but they are worsened by adiposity. Endothelial dysfunction is a condition predisposing to atherosclerosis and is the result of the interplay of CMR factors, overall high levels of total and low-density lipoprotein (LDL) cholesterol. Instead, increased adiposity seems to be a strong predictor of vascular impairment, involving abdominal aorta before any other vascular structure (14).

#### Hypercoagulable State

Obesity causes early endothelial damage since childhood, when high levels of von Willebrand factor (vWF) and plasminogen activator inhibitor (PAI)-1 antigens, well-known markers of endothelial dysfunction, may be detected. High PAI-1 levels cause a state of hypofibrinolysis, favoring recurrent microthrombi. The result is a hypercoagulable state, with high levels of thrombin–antithrombin complex, D-dimer, and fibrinogen, expression of increased thrombin generation (15) and risk of thromboembolic events, especially in adolescents (16). Furthermore, clots from obese children have significantly longer lysis time than control clots to tissue plasminogen activator (t-PA)-induced fibrinolysis. Thrombin activatable fibrinolysis inhibitor (TAFI) is higher as well and likely contributes to obesity-related fibrinolysis resistance. Thrombin can be considered its major activator, so that high levels of TAFI are likely the consequence of enhanced thrombin generation (17).

Kohorst et al. (18) demonstrated that sedentary behaviors, such as playing videogames, are additional prothrombotic risk factors associated with obesity, remarking the importance of lifestyle.

#### Inflammation

Systemic inflammation plays a major role in the development of coronary artery disease in childhood obesity. Adipose tissue, mainly abdominal fat, acts as an endocrine and a paracrine organ, releasing hormones, growth factors, and proinflammatory cytokines [leptin, resistin, adiponectin, interleukin (IL)-6, tumor necrosis factor (TNF)-α, high-sensitivity C-reactive protein (hs-CRP)]. They support chronic inflammation, enhance the development and/or the progression of chronic diseases, and play a role in the athero-inflammation physiology (19). High levels of leptin and reduced adiponectin concentrations (pro- and anti-inflammatory adipokines, respectively) are observed in obese adolescents.

The role of hs-CRP has been largely investigated. It seems involved in the initial phase of atherosclerosis, facilitating adhesion and migration of monocytes into the arterial wall and altering the vascular reactivity by the inhibition of *in vitro* nitric oxide synthesis. Its levels in the pediatric population are predictive of CVDs and may be routinely assessed in clinical practice to stratify the risk for coronaropathies, in addition to traditional factors such as blood pressure (BP) and cholesterol (20). Interestingly, a lifestyle intervention on obese adolescents affects the body composition and may reduce the hs-CRP levels, decreasing CVD risk (21).

#### Blood Pressure Effects

Hypertension in adolescence is an important predictor of later endothelial dysfunction, and thus a correct evaluation and follow-up of BP are mandatory. Hypertension is defined as systolic and/or diastolic BP ≥ 95th percentile for gender, age, and height on at least three occasions (22). Garcia-Espinosa et al. (23) studied the relationship between BMI and different cardiovascular parameters, such as peripheral and aortic BP, aortic wave-derived parameters, common carotid, femoral, and brachial artery diameters and stiffness, carotid intima–media thickness (cIMT), and carotid–radial and carotid–femoral pulse wave velocity. They showed that BMI is independently associated with hemodynamic and arterial parameters, but not with diastolic BP, which presents the major variations in association with BMI. On the contrary, systolic BP has the highest regression coefficient among all the hemodynamic parameters with a variation of ~2, 5, or 7 mmHg for BMI variation of 1, 2, or 3 SDS, respectively (23).

Along with hypertension, obesity induces a decrease in systemic peripheral resistance, which yields hyperdynamic circulation. These changes may induce alterations and remodeling of the large vessels and the heart. Structural modifications in the arterial wall may be studied, since childhood (24), on ultrasound with IMT, which is affected by the duration and the severity of obesity and is a well-known marker of subclinical atherosclerosis and CVDs. In obese children, the compliance of the greater arteries (carotid arteries, aorta, and pulmonary artery) is decreased, while cIMT, arterial stiffness, and prevalence of the presence of coronary artery calcium are increased.

### Heart Damage

The vascular changes increase the ventricular afterload. High fat mass is associated with increased activation of the renin–angiotensin–aldosterone system and sympathetic tone, increasing intravascular volume and consequently ventricular preload. Hyperdynamic circulation leads to dilatation and hypertrophy of the left ventricle. A recent study (25) showed that cardiac remodeling could occur in obese children, with a larger left ventricular mass index. In this study, 24% of patients had concentric hypertrophy, which causes an alteration of contractile function and is the type of cardiac remodeling most closely related to mortality. Impaired circumferential and longitudinal strains were demonstrated in obese children, causing diastolic dysfunction.

**Figure 1:**
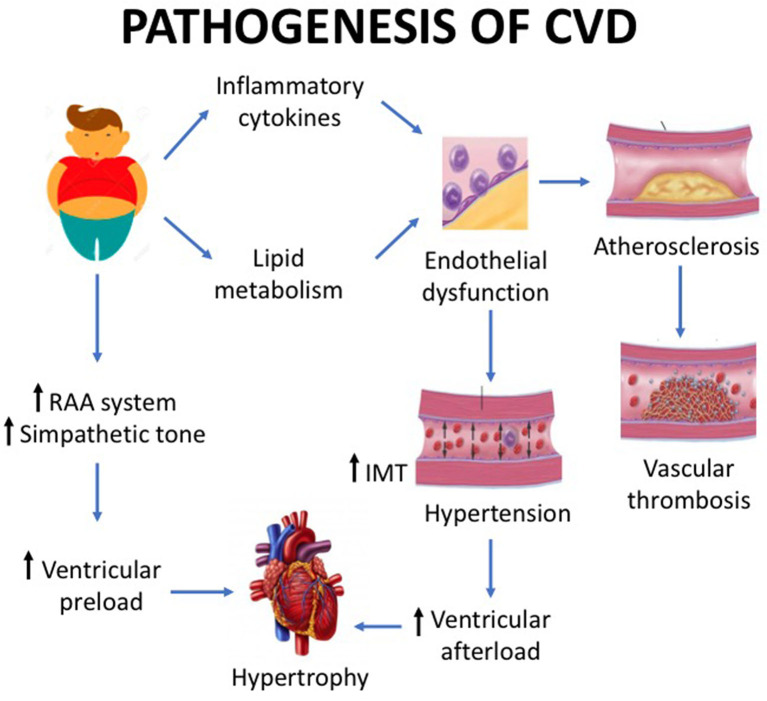
Childhood obesity is associated with a number of cardiovascular structural and functional alterations. Basically, the pathogenesis of cardiovascular disease is caused by the increase in preload and by the vascular damage. The preload increase is mediated by the renin–angiotensin system, while the vascular damage by lipid metabolism impairment and pro-inflammatory cytokine pathway. The latter mechanisms cause endothelial dysfunction, which in turn leads to hypertension with ventricular afterload and endothelial damage with subsequent atherosclerosis. The effect of preload increase and vascular damage is myocardial hypertrophy.

Echocardiography can be used to study ventricular function, but in obese patients with normal heart by traditional observation, tissue Doppler echocardiography may be used to show ventricular dysfunction. This technique showed that obese children have an earlier cardiac impairment (systolic and diastolic dysfunctions) compared to healthy controls (26). The effects on cardiac structure and function persist into adulthood and contribute to increase the risk of CVDs and mortality. Fortunately, the increased ventricular strain is reversible, and obese children, normal weight in adulthood, has a CVD risk comparable to persons who have never been obese (27). Figure 1 displays the mechanisms underlying myocardial hypertrophy.

Obesity is a risk factor for sudden death and is associated with delayed ventricular repolarization. Mild lengthening of the QTc interval and increase in QT or QTc dispersion are reported, but it is not clear if QT alterations are caused by obesity *per se* or by factors that directly affect this process. Moreover, this finding would be reversible with weight loss (28).

### Metabolic Consequences

Visceral fat secretes adipokines, such as adiponectin, leptin, monocyte chemoattractant protein (MCP)-1, resistin, retinol binding protein (RBP)-4, and ILs, which enhance the development and/or the progression of chronic diseases, overall insulin resistance. Visceral fat increases turnover of free fatty acids, which facilitate insulin resistance, first step toward T2D. The risk is as higher as higher BMI and longer duration of obesity (29).

It was shown that youth obesity correlates positively with BP and triglycerides but negatively with high-density lipoprotein (HDL) cholesterol in adulthood (30). Zabarsky et al. (31) reported in 2,244 obese children, divided into four groups according to obesity degrees, that LDL cholesterol was comparable among the obesity categories, while HDL cholesterol tends to decrease and triglycerides to increase, raising the cardiovascular risk. The risk is further increased in males, as the prevalence of high-risk lipid levels is higher than in females irrespective of BMI and WC (32).

Obesity plays a key role in the development of MetS, a complex picture characterized by a combination of risk factors, such as WC, triglycerides, HDL cholesterol, BP, and glucose. The definition of pediatric MetS requires age- and sex-based standards and is not universally accepted (Supplementary Table 2). Youth obesity is strongly associated with adulthood MetS, and this association determines that childhood obesity and MetS are significant predictors of CMR in adulthood. Age plays a pivotal role: adulthood MetS is predicted by childhood MetS from the age of 5 years, while a significant relation for T2D and subclinical atherosclerosis begins between 8 and 14 years of age (33).

## Syndromic Obesity

In this part, we reviewed the papers about CVDs in syndromic obesity. Syndromic obese patients develop cardiovascular consequences, but they often present metabolic peculiarities and additional congenital heart problems. They present intellectual disability, making primary prevention very difficult.

### Prader–Willi Syndrome

Obesity is the major cause of morbidity and mortality, and most Prader–Willi Syndrome (PWS) adults feature cardiovascular and metabolic diseases such dyslipidemia, atherosclerosis, diabetes, and MetS. Defects in the hypothalamic pathway of satiety control lead to hyperphagia. Fat mass is higher and lean mass lower than weight-matched subjects with idiopathic obesity, making BMI less reliable than other indexes of central adiposity to assess CMR.

In PWS, high ghrelin levels correlate with cIMT, suggesting a role as a potential risk factor for atherosclerosis and coronaropathies. The ghrelin–obestatin ratio changes during development (obestatin and ghrelin originate from the same precursor), decreasing from early to late childhood in parallel with pancreatic insulin secretion. This decline seems correlated with glucose derangement (34), which is much more frequent in obese patients and in adult patients rather than in childhood and normal-weight patients (35).

PWS patients present high hs-CRP levels, which correlate with atherosclerosis and probably with coronary disease causing early sudden death. Plasma hs-CRP increases with age, suggesting that chronic inflammation increases the CVD risk in adulthood.

Obesity plays a key role in the development of MetS. Its severity depends on body fat mass and is more frequent in obese than in nonobese PWS, irrespective of age, suggesting an independent role in the metabolic risk. Additional researches are needed to understand the relationship between MetS and PWS, and all mechanisms involved (36). On the contrary, PWS seems to play by itself a protective role on the cardiometabolic profile: obese PWS patients present better glucose tolerance, higher HDL cholesterol, and lower BMI than non-syndromic obese youths after standardization for fat mass.

Close nutritional monitoring is mandatory, but pharmacological approach to obesity would be optimal. Glucagon-like peptide (GLP)-1 receptor agonists seem effective in reducing the CMR (37).

### Alström Syndrome

Alström syndrome (ALMS) patients may present an increased and diffuse interstitial myocardial fibrosis and abnormalities of left ventricular function, leading to cardiomyopathy (38). Isolated severe dilated cardiomyopathy as first symptom with premature exitus was reported (39, 40). The pathophysiology of cardiovascular morbidity and mortality is likely different between childhood and adulthood, when a history of infantile cardiomyopathy does not appear to heighten the risk of adverse cardiovascular outcomes. The cardiac fibrosis seems actively modulated by the severity of metabolic dysfunction, with elevated triglycerides that act as intermediary or potential marker (41).

Obesity is the major cause of early development of T2D, whose duration is directly proportional to the increase of aortic pulse wave velocity, predicting in turn cardiovascular events (42).

### Bardet–Biedl Syndrome

Cardiomyopathy in Bardet–Biedl syndrome (BBS) is less frequent than in ALMS, but some cases are reported with unclear etiological mechanisms (43). Tricuspid and pulmonary valve defects and stenosis are described, suggesting that cilia may play a role also in valve development (mitral, tricuspid, and pulmonary), despite the genetic mechanisms are not yet understood (44).

### Carpenter Syndrome

Carpenter syndrome may feature congenital heart disease, such as ventricular septal defect, patent ductus arteriosus, pulmonic stenosis, Fallot tetralogy and laterality defects, likely related to a particular mutation identified in the multiple epidermal growth factor-like domains 8. Cardiac complications are associated with rare cases of sudden death, but data are insufficient to speculate on any possible mechanisms (45).

### Cohen Syndrome

The mechanisms of cardiovascular and metabolic disorders remain unclear in this syndrome. Notwithstanding, valvular and vascular defects, essential and pulmonary hypertension have been reported (46). These patients are prone to present criteria for MetS and an increased risk of T2D (47).

### Fragile X Syndrome

The rate of abnormal aortic root dimensions and mitral valve prolapse is higher in Fragile X syndrome (FXS) than in the general population (48). Adipokines seem to be involved not only in the metabolic consequences but also in the psychiatric features of these patients. Lisik et al. (49) investigated their levels in young adult patients, showing that leptin levels are significantly higher and adiponectin levels significantly lower than in healthy controls. Furthermore, adiponectin concentrations showed a moderate correlation with age, while leptin concentrations with BMI.

A link between vascular regulation and neuronal and non-neuronal abnormalities is represented by vascular endothelial growth factor (VEGF)-A. Blocking VEGF-A can alleviate FXS abnormalities, but a possible link between cardiovascular consequences of obesity and neuronal and non-neuronal abnormalities is yet to be ascertained (50, 51).

FXS patients display a trend toward lower cholesterol and triglyceride levels than healthy people (49), and the trend is more evident in males than in females (52). The FMR1 gene encodes fragile X mental retardation protein, whose absence leads to FXS phenotype and autism. The absence of this protein affects the liver function and the lipid homeostasis, enhancing glucose tolerance and insulin response and reducing adiposity (53). This mechanism likely accounts for the features of lipid metabolism.

Severe obesity is not so frequent as in other syndromic obesity forms, but in the case of obesity, the phenotype is PWS-like. In the case of insulin resistance/T2D, metformin is effective and interestingly beneficial also on language, cognition, and behavior problems such as overeating, irritability, social avoidance, and aggression (54, 55).

## Idiopathic and Syndromic Obesity: Pathway In Cardiovascular Implications

Obese children are prone to early development of CVD risk factors such as insulin resistance, dyslipidemia, vascular changes, and hypertension since adolescents. Visceral fat secretes adipokines (which enhance the development of insulin resistance) and increases both the turnover of free fatty acids (which facilitate insulin resistance) and high-risk lipid levels (which increase the risk for CVDs). Endothelial dysfunction plays a pivotal role in atherogenesis and other CVDs, which can be precociously identified by measuring blood pressure, inflammation, and hypercoagulable state markers. Blood flow changes may induce alterations and remodeling of large vessels and heart. This pathway leads to early cardiovascular events during adulthood, but in children with syndromic obesity, these mechanisms are modulated by specific features that may predispose to or protect from cardiovascular complications. The distribution of lean and fat mass between patients with idiopathic and syndromic obesity is different, and thus the prognostic role of anthropometric markers is different. The former are prone to develop metabolic risk factors for CVDs earlier than the latter. Patients with BBS, ALMS, Carpenter syndrome, and Cohen syndrome may present heart anomalies, which actively modulate the development of hearth complications and increase the risk of sudden death. PWS patients present higher inflammation status and early glucose derangement, which worsen with age and accelerate the onset of CVDs. Similarly, FXS patients have higher pro-inflammatory cytokines that increase with age and BMI, worsening the prediction of CVDs. PWS and FXS seem independent protective factors for the cardiometabolic profile and may be effectively treated with specific drugs.

Table 1 compares the mechanisms leading to cardiovascular implications in idiopathic and syndromic obesity, along with inheritance and phenotype.

**Table 1:** The table compares the most important pathophysiological mechanisms leading to cardiovascular consequences of idiopathic obesity and of each syndrome described in the paper.

**Disorder**	**Molecular genetics**	**Clinical Features**	**Cardiometabolic Mechanisms**
Idiopathic obesity	Multifactorial disease Increasing evidence that genetic variants interact with environmental factors through epigenetic regulation	Chronic metabolic disorder associated with increased morbidity and mortality Dyslipidemia, hypertension, stroke, insulin resistance, but also orthopedic, oncological, psychological, respiratory and other comorbidities associated with increased body weight	Endothelial dysfunction and great vessels impairment linked with: a. hypercoagulable state, prone to thromboembolism; b. chronic inflammation; c. hypertension and hyperdynamic circulation; d. left ventricle dilatation and hypertrophy with reversible cardiac remodeling; e. insulin and other MetS risk factors
Prader-Willi Syndrome (PWS)	Loss of function of specific genes on the paternally inherited 15q11.2-q13 chromosomal region due to paternal gene deletion or maternal uniparental disomy 15 or imprinting defects	Most common cause of syndromic obesity Hypogonadism, intellectual disability, short stature, acromicria, low birth weight, hypotonia, feeding difficulties, followed in later infancy by hyperphagia and gradual development of severe obesity; low lean mass, high fat mass	Ghrelin is a potential marker of atherosclerosis Chronic inflammation Visceral fat is associated with frequency and severity of MetS Apparent protective role on the cardiometabolic profile High risk of T2D GLP-1 receptors agonists significantly decrease blood glucose and seem to reduce WC and BMI
Alström syndrome (ALMS)	Autosomal recessive disease, characterized by defects in ALMS1 gene on chromosome 2p13 ALMS1 is a ubiquitous protein whose function is not yet fully known	Retinitis pigmentosa and cone-rod dystrophy (100% of patients); neurosensory deafness (70% of patients); obesity (100% of patients) with dyslipidemia, hypertension, hyperinsulinemia and consequent progression to T2D	Myocardial fibrosis and left ventricular dysfunction MetS worsens cardiac fibrosis Early obesity-related T2D onset, which affects the CVD risk
Bardet-Biedl syndrome (BBS)	Autosomal recessive disease, caused by defects in different BBS genes	Similar to ALMS Cone-rod dystrophy (93–100%), obesity (72–88%), and adrenal abnormalities (25–100%). BBS differs from ALMS for cognitive impairment and polydactyly	Congenital heart defects (cardiomyopathy, tricuspid and pulmonary valve defects and stenosis)
Carpenter Syndrome	Carpenter syndrome-1 (CRPT1) is an autosomal recessive disease, caused by homozygous mutation in the RAB23 gene on chromosome 6p11 Carpenter syndrome-2 (CRPT2) is an autosomal recessive disease, caused by mutation in the MEGF8 gene and is characterized by the association between features of CRPT2 and defective lateralization	Acrocephaly, polysyndactyly, frequent obesity, intellectual disability, umbilical hernia, cryptorchidism and congenital heart disease CRPT2 is characterized by the association of features of CRPT2 and defective lateralization	Congenital heart defects (sept defect, patent ductus arteriosus, pulmonic stenosis, Fallot tetralogy) in CRPT2
Cohen Syndrome	Autosomal recessive disease, caused by mutations in the COH1 gene on chromosome 8q22	Obesity, hypotonia, intellectual disability and craniofacial anomalies	Valvular and vascular defects Essential and pulmonary hypertension Prone to develop MetS and T2DM
Fragile X Syndrome (FXS)	X-linked disease, caused by the expansion mutation of a CGG repeat sequence in the FMR1 gene, encoding for a protein essential for synaptic plasticity, neuronal morphology, and cognitive development.	Most common genetic cause of inherited intellectual disabilities and autism spectrum disorders Cognitive dysfunction, hyperactivity, impulsivity, communication problems, and autism spectrum disorders PWS like obesity, prominent forehead, narrow face, protruding ears, high-arched palate, strabismus, pectus excavatum, macroorchidism	Trend to lower cholesterol and triglycerides levels PWS-like obesity Adipokines affect metabolic profile and psychiatric features Metformin beneficial both on the metabolic profile and the neurological features

*Inheritance and clinical features are reported as well*.

## Conclusion

Obese children have a shorter life expectation than normal-weight age-matched controls, and thus primary prevention is mandatory. Obesity is considered as a chronic disease playing a key role in the pathogenesis of long-term life-threatening diseases, mostly CVDs, mediated by metabolic and cardiovascular risk factors. Patients with syndromic obesity feature additional mechanisms that exert independent roles on the CMR, but we have few data and definitive conclusions deserve caution. Primary prevention is the primary goal to reduce the burden for health, but it is much more difficult for syndromic patients. More studies in larger cohorts would be appreciated to unravel the mechanisms underlying the cardiovascular consequences and to identify any possible target for the pharmacological approach of obesity and its related disorders.

## Author Contributions

MD and PG equally contributed to the concept and design of the review and reviewed and approved the submitted manuscript. CP and MD performed the literature search. CP and FV evaluated the papers and wrote the draft of the manuscript.

## Conflict of Interest

The authors declare that the research was conducted in the absence of any commercial or financial relationships that could be construed as a potential conflict of interest.

## Supplementary Material

Supplementary Figure 1 Childhood obesity causes adult obesity, and metabolic and cardiovascular disease in childhood. In adults, the risk of cardiovascular disease is affected by obesity and cardiovascular disease in childhood. On the other hand, the risk of metabolic disorders is affected by obesity and metabolic disorders in childhood.

Supplementary Table 1 The table displays the etiology of childhood obesity. Modified by Aggarwal et al. (6).

Supplementary Table 2 The table summarizes the classification of metabolic syndrome at different age. DBP, diastolic blood pressure; MetS, metabolic syndrome; SBP, systolic blood pressure; T2D, type 2 diabetes mellitus. Modified by Nehus and Mitsnefes (2).
