# Supplementary figures and images for: Cardiovascular Implications in Idiopathic and Syndromic Obesity in Childhood: An Update
Source: Front Endocrinol (Lausanne). 2020 Jun 9;11:330. doi: 10.3389/fendo.2020.00330 (PMC7296059; doi:10.3389/fendo.2020.00330)

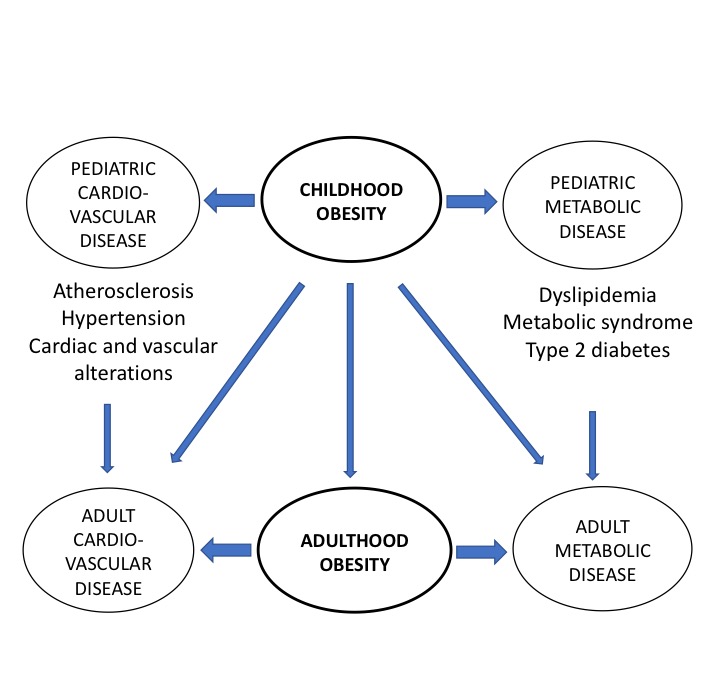

Supplement: Supplementary Figure 1 — Childhood obesity causes adult obesity, and metabolic and cardiovascular disease in childhood. In adults, the risk of cardiovascular disease is affected by obesity and cardiovascular disease in childhood. On the other hand, the risk of metabolic disorders is affected by obesity and metabolic disorders in childhood. [file Image_1.JPEG]
