# Supplementary material for: Cardiovascular Implications in Idiopathic and Syndromic Obesity in Childhood: An Update
Source: Front Endocrinol (Lausanne). 2020 Jun 9;11:330. doi: 10.3389/fendo.2020.00330 (PMC7296059; doi:10.3389/fendo.2020.00330)
Supplement: Supplementary Table 1 — The table displays the etiology of childhood obesity. Modified by Aggarwal et al. (6). [file Table_1.docx]

**Supplementary Table 1. Etiology of Childhood Obesity**

| **Exogenous obesity** |
| --- |
| • Chronic imbalance between energy intake and expenditure: |
| Increased intake of refined carbs and sugars; Sedentary lifestyle (watching tv, playing video games). |
| • Medications: |
| Glucocorticoids, tricyclic antidepressants, risperidone |
| • Adverse metabolic programming (acts in conjunction with diet and lifestyle factors) |
| Small for gestational age (SGA);  Large for gestational age (LGA);  Maternal obesity or diabetes. |
| **Endogenous obesity** |
| • Monogenic causes: |
| Defects in genes encoding melanocortin 4 receptor (MC4R), leptin (LEP),  leptin receptor (LEPR), pro-opiomelanocortin (POMC) etc. |
| • Genetic syndromes: |
| Alstrom, Bardet- Biedl, Prader Willi, Beckwith- Wiedemann, Carpenter, Cohen,  Albright Hereditary Osteodystrophy etc. |
| • Endocrine causes: |
| Hypothyroidism, Cushing syndrome, hypothalamic obesity, growth hormone deficiency etc. |

| **Supplementary table 2. Criteria for diagnosis of childhood and adolescent MS** | | | | | |
| --- | --- | --- | --- | --- | --- |
| **Age Group** | **Waist Circumference** | **Triglycerides** | **HDL-Cholesterol** | **Blood Pressure** | **Glucose** |
| 6-10 years | ≥ 90^th^  percentile | Insufficient data (MS cannot be diagnosed) | | | |
| 10–16 years |  | ≥ 150mg/dL | < 40mg/dL | SBP≥130 mmHg  or  DBP≥85 mmHg | ≥100 mg/dL  or  known T2DM |
| >16 years | ≥ 94 cm (M)  ≥ 80 cm (F) |  | < 40mg/dL (M)  < 50mg/dL (F) |  |  |
