# Supplementary material for: Cardiovascular Implications in Idiopathic and Syndromic Obesity in Childhood: An Update
Source: Front Endocrinol (Lausanne). 2020 Jun 9;11:330. doi: 10.3389/fendo.2020.00330 (PMC7296059; doi:10.3389/fendo.2020.00330)
Supplement: Supplementary Table 2 — The table summarizes the classification of metabolic syndrome at different age. DBP, diastolic blood pressure; MetS, metabolic syndrome; SBP, systolic blood pressure; T2D, type 2 diabetes mellitus. Modified by Nehus and Mitsnefes (2). [file Table_2.docx]

| **Disorder** | **Molecular genetics** | **Clinical Features** | **Cardiometabolic Mechanisms** |
| --- | --- | --- | --- |
| **Idiopathic obesity** | Multifactorial disease  Increasing evidence that genetic variants interact with environmental factors through epigenetic regulation | Chronic metabolic disorder associated with increased morbidity and mortality  Dyslipidemia, hypertension, stroke, insulin resistance, but also orthopedic, oncological, psychological, respiratory and other comorbidities associated with increased body weight | Endothelial dysfunction and great vessels impairment linked with: a. hypercoagulable state, prone to thromboembolism; b. chronic inflammation; c. hypertension and hyperdynamic circulation; d. left ventricle dilatation and hypertrophy with reversible cardiac remodeling; e. insulin and other MetS risk factors |
| **Prader-Willi**  **Syndrome (PWS)** | Loss of function of specific genes on the paternally inherited 15q11.2-q13 chromosomal region due to paternal gene deletion or maternal uniparental disomy 15 or imprinting defects | Most common cause of syndromic obesity  Hypogonadism, intellectual disability, short stature, acromicria, low birth weight, hypotonia, feeding difficulties, followed in later infancy by hyperphagia and gradual development of severe obesity; low lean mass, high fat mass | Ghrelin is a potential marker of atherosclerosis  Chronic inflammation  Visceral fat is associated with frequency and severity of MetS  Apparent protective role  on the cardiometabolic profile  High risk of T2D  GLP-1 receptors agonists significantly decrease blood glucose and seem to reduce WC and BMI |
| **Alström**  **syndrome (ALMS)** | Autosomal recessive disease, characterized by defects in ALMS1 gene on chromosome 2p13  ALMS1 is a ubiquitous protein whose function is not yet fully known | Retinitis pigmentosa and cone-rod dystrophy (100% of patients); neurosensory deafness (70% of patients); obesity (100% of patients) with dyslipidemia, hypertension, hyperinsulinemia and consequent progression to T2D | Myocardial fibrosis and left ventricular dysfunction  MetS worsens cardiac fibrosis  Early obesity-related T2D onset, which affects the CVD risk |
| **Bardet-Biedl**  **syndrome (BBS)** | Autosomal recessive disease, caused by defects in different BBS genes | Similar to ALMS  Cone-rod dystrophy (93-100%), obesity (72-88%), and adrenal abnormalities (25-100%). BBS differs from ALMS for cognitive impairment and polydactyly | Congenital heart defects (cardiomyopathy, tricuspid and pulmonary valve defects and stenosis) |
| **Carpenter**  **Syndrome** | Carpenter syndrome-1 (CRPT1) is an autosomal recessive disease, caused by homozygous mutation in the RAB23 gene on chromosome 6p11  Carpenter syndrome-2 (CRPT2) is an autosomal recessive disease, caused by mutation in the MEGF8 gene and is characterized by the association between features of CRPT2 and defective lateralization | Acrocephaly, polysyndactyly, frequent obesity, intellectual disability, umbilical hernia, cryptorchidism and congenital heart disease  CRPT2 is characterized by the association of features of CRPT2 and defective lateralization | Congenital heart defects (sept defect, patent ductus arteriosus, pulmonic stenosis, Fallot tetralogy) in CRPT2 |
| **Cohen**  **Syndrome** | Autosomal recessive disease, caused by mutations in the COH1 gene on chromosome 8q22 | Obesity, hypotonia, intellectual disability and craniofacial anomalies | Valvular and vascular defects  Essential and pulmonary hypertension  Prone to develop MetS and T2DM |
| **Fragile X**  **Syndrome (FXS)** | X-linked disease, caused by the expansion mutation of a CGG repeat sequence in the FMR1 gene, encoding for a protein essential for synaptic plasticity, neuronal morphology, and cognitive development. | Most common genetic cause of inherited intellectual disabilities and autism spectrum disorders  Cognitive dysfunction, hyperactivity, impulsivity, communication problems, and autism spectrum disorders  PWS like obesity, prominent forehead, narrow face, protruding ears, high-arched palate, strabismus, pectus excavatum, macroorchidism | Trend to lower cholesterol and triglycerides levels  PWS-like obesity  Adipokines affect metabolic profile and psychiatric features  Metformin beneficial both on the metabolic profile and the neurological features |
